# Role of non-coding RNAs in tumor progression and metastasis in pancreatic cancer

**DOI:** 10.1007/s10555-021-09995-x

**Published:** 2021-09-30

**Authors:** Lorenzo F. Sempere, Katherine Powell, Jatin Rana, Andrew A. Brock, Thomas D. Schmittgen

**Affiliations:** 1 Precision Health Program, College of Human Medicine, Michigan State University, East Lansing, MI 48824 USA; 2 Department of Medicine, College of Human Medicine, Michigan State University, East Lansing, MI 48824 USA; 3 Department of Pharmaceutics, College of Pharmacy, University of Florida, Gainesville, FL 32610 USA

**Keywords:** microRNA (miR, miRNA), Long non-coding RNAs (lncRNA), Circular non-coding RNA (circRNA), Extracellular vesicles

## Abstract

Pancreatic ductal adenocarcinoma (PDAC) is one of the most lethal types of cancer with an overall 5-year survival rate of less than 10%. The 1-year survival rate of patients with locally advanced or metastatic disease is abysmal. The aggressive nature of cancer cells, hypovascularization, extensive desmoplastic stroma, and immunosuppressive tumor microenvironment (TME) endows PDAC tumors with multiple mechanisms of drug resistance. With no obvious genetic mutation(s) driving tumor progression or metastatic transition, the challenges for understanding the biological mechanism(s) of these processes are paramount. A better understanding of the molecular and cellular mechanisms of these processes could lead to new diagnostic tools for patient management and new targets for therapeutic intervention. microRNAs (miRNAs) are an evolutionarily conserved gene class of short non-coding regulatory RNAs. miRNAs are an extensive regulatory layer that controls gene expression at the posttranscriptional level. This review focuses on preclinical models that functionally dissect miRNA activity in tumor progression or metastatic processes in PDAC. Collectively, these studies suggest an influence of miRNAs and RNA-RNA networks in the processes of epithelial to mesenchymal cell transition and cancer cell stemness. At a cell-type level, some miRNAs mainly influence cancer cell–intrinsic processes and pathways, whereas other miRNAs predominantly act in distinct cellular compartments of the TME to regulate fibroblast and immune cell functions and/or influence other cell types’ function via cell-to-cell communications by transfer of extracellular vesicles. At a molecular level, the influence of miRNA-mediated regulation often converges in core signaling pathways, including TGF-β, JAK/STAT, PI3K/AKT, and NF-κB.

## Introduction

Pancreatic ductal adenocarcinoma is one of the most lethal types of cancer with an overall 5-year survival rate of less than 10% [1]. Virtually all long-term survivors are patients with early stage disease for whom surgical resection and adjuvant systemic therapy provides a potential cure [2, 3]. Due to late clinical presentation, about 30% of patients are diagnosed with locally advanced disease and about 50% of patients with metastatic disease [2]. The 1-year survival rate of these advanced stage cases is abysmal [2, 3]. Modifications in systemic chemotherapy regimens such as FOLFIRINOX (5-fluorouracil, folinic acid, irinotecan, and oxaliplatin) or gemcitabine-containing combination treatments have resulted in a modest improvement of patient outcome in recent years [2]. Targeted therapies and immunotherapies, which have improved outcome in other cancer types, have failed to provide a clear clinical signal in PDAC [2, 4]. The aggressive nature of cancer cells, hypovascularization, extensive desmoplastic stroma, and immunosuppressive tumor microenvironment (TME) endows PDAC tumors with multiple mechanisms of drug resistance [3, 5, 6]. Combination strategies that target cancer cells and elements of the TME may offer new opportunities and hope in PDAC. Myeloid cell-modifying agonist CD40 antibody, stroma-modifying angiotensin-receptor blocker Losartan, CXCR4 antagonist in combination with PD-1 blockade, and connective tissue growth factor inhibitors are showing promising results in on-going clinical trials [2, 7, 8]. In addition, recent studies continue to investigate the clinical value of circulating tumor DNA and other molecular biomarkers for early disease detection [9, 10].

PDAC has a limited number of driver genes, with frequent and predominant mutations in *KRAS* (> 90%), *CDKN2A* (~ 90%), *TP53* (~ 70%), and *SMAD4* (~ 55%) [3, 11]. With no obvious genetic mutation(s) driving tumor progression or metastatic transition, the challenges for understanding the biological mechanism(s) of these processes are paramount. These require a comprehensive and integrative analysis of dynamic changes in gene dosage, transcriptome, epigenome, core signaling, and metabolomic pathways that collectively contribute to tumor evolution and metastatic spread [11–15]. A better understanding of the molecular and cellular mechanisms of these processes could lead to new diagnostic tools for patient management and new targets for therapeutic intervention. microRNAs (miRNAs) are an evolutionarily conserved gene class of short non-coding regulatory RNAs [16–18]. miRNAs are an extensive regulatory layer that controls gene expression at the posttranscriptional level. miRNA expression and function has been linked to different aspects of PDAC biology and disease progression [18–23]. This review focuses on preclinical models that functionally dissect miRNA activity in tumor progression or metastatic processes. Collectively, these studies suggest an influence of miRNAs and RNA-RNA networks in the processes of epithelial to mesenchymal cell transition (EMT), proliferation, and cancer cell stemness. At a cell-type level, some miRNAs mainly influence the processes and pathways in a cancer cell–intrinsic manner, whereas other miRNAs predominantly act in distinct cellular compartments of the TME to regulate fibroblast and immune cell functions and/or to dictate other cell types’ functions via cell-to-cell communication by transfer of extracellular vesicles (EVs). At a molecular level, the influence of miRNA-mediated regulation often converges in core signaling pathways, including TGF-β, JAK/STAT, PI3K/AKT, and NF-κB. In the following sections, we discuss salient examples of these mechanisms of action and crosstalk between cancer cells and other cell types in the TME. We focus our discussion on recent studies in which there is an *in vivo* demonstration of miRNA activity in tumor progression and/or metastatic processes. This *in vivo* demonstration may involve the use of genetically engineered mouse models, organoid or cell line xenograft models, and/or patient-derived tumor models.

## microRNA biogenesis and dysregulation during PDAC progression

**Fig. 1:**
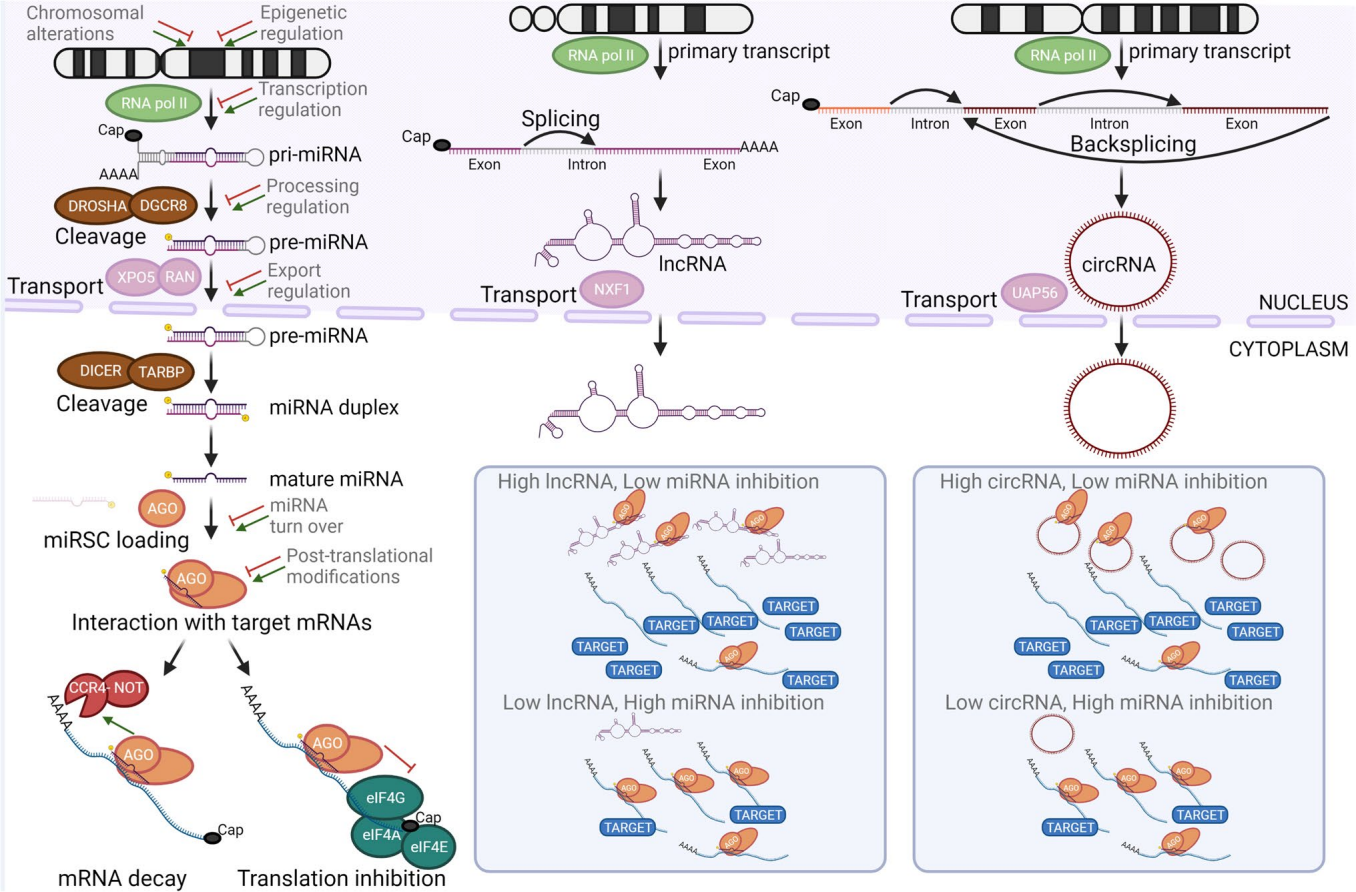
Biogenesis of miRNAs and other non-coding RNAs. Key steps of biogenesis of miRNAs and miRNA-interacting lncRNAs and circRNAs. Several mechanisms are known that affect regulation of miRNA expression and/or activity. Nuclear export is key step for cytoplasmic interaction of miRNAs and these other classes of ncRNAs that sequester miRNAs away from target mRNAs and indirectly increase protein product of the target gene. Abbreviations: AGO, argonaute RISC component; circRNA, circular non-coding RNA; CCR4-NOT, carbon catabolite repression-negative on TATA-less complex; DICER, ribonuclease III Dicer1; DGRCR8, DiGeorge syndrome critical region gene 8, microprocessor complex subunit; DROSHA, ribonuclease III Dicer1; eiF4, eukaryotic translation initiation factor 4; lncRNA, long non-coding RNA; miRISC, miRNA-induced silencing complex; NXF1, nuclear RNA export factor 1; RAN, member of RAS oncogene family, small nuclear GTPase; RNA pol II, RNA polymerase II; miRNA, microRNA; TARBP, TAR (HIV-1) RNA-binding protein 1; UAP56, U2AF65-associated protein 56 also known as DDX39B (DExD-box helicase 39B); XPO5, exportin 5

The mature and biological active miRNA consists of a 19–24 nucleotide-long RNA molecule resulting from a stepwise processing of a much longer mRNA-like primary transcript (Fig. 1). The immense majority of the mRNA-like primary transcripts are transcribed by RNA polymerase II [16, 17]. Some miRNAs are arranged in tightly linked gene clusters, and this primary transcript (pri-miRNA) may contain the precursor hairpin RNA (pre-miRNA) for several miRNAs [24]. Transcription of either a single miRNA or a miRNA gene cluster may initiate from a proximal promoter to the miRNA gene or distal promoter driving expression of the host gene [16, 17]. In the nucleus, RNAse III DROSHA-containing RNA microprocessor complex cleaves precursor hairpin RNA that is exported to the cytoplasm by XPO5-facililated pathway [17]. In the cytoplasm, another RNAse III DICER cleaves the final mature RNA molecule that becomes functionally active when loaded into the ARGONAUTE-containing miRNA-induced silencing complex (miRISC). The miRNA serves as a guide to direct miRISC in close proximity to target mRNA by binding to partially complementary sites, typically on the 3′ UTR. The interaction of miRISC with other mRNA-bound protein complexes (e.g., CCR4-NOT, eIF4A-G) leads to mRNA decay, mRNA cleavage, and/or inhibition of translation initiation that ultimately results in a decreased protein output of the target gene(s) [16, 17]. In most cases, miRNA binding to the mRNA is driven by the seed region (2^nd^ to 8^th^ nucleotides of the miRNA) which allows for interactions of a single miRNA with tens or hundreds of mRNAs. The relative abundance of miRNA to target mRNA and number of binding sites and binding affinity determines the extent of protein output reduction for each target gene [18]. A single miRNA can influence cellular programs by profound downregulation of a handful of key targets and/or by more modest but coordinated downregulation of a larger number of target genes. About 2,000 *bona fide* miRNA genes have been identified in the human genome [24]; it is estimated that all miRNAs expressed in a particular cell can modulate the expression of up to 60% of the protein-encoding genes and correspondingly influence a large number of cellular programs [16, 17, 25].

Dysregulation of miRNA expression and activity in PDAC can occur at different levels from chromosomal alteration (e.g., gain or loss of copy numbers), epigenetic and transcriptional regulation of the pri-miRNA, processing of pri-miRNA, export and processing of pre-miRNA, and/or physical interaction with other non-coding RNAs (Fig. 1). Dysregulation of miRNA expression has been extensively characterized between normal pancreas and PDAC tumor specimens, chronic pancreatitis and PDAC tumor specimens, and PDAC precursor lesions and PDAC tumor specimens at different clinical stages. These tissue correlative studies have led to the identification of specific miRNAs or miRNA signatures that could serve as diagnostic and/or prognostic biomarkers [18, 20, 21]. While there is no complete overlap among different studies, altered expression of miRNAs (e.g., let-7a, miR-10b, miR-21, miR-217) has been frequently associated with disease progression and/or metastatic disease. Guided by these clinical observations, many of these miRNAs have been subjected to a battery of *in vitro* and *in vivo* functional assays to dissect the etiological contribution of each of these miRNAs to a specific metastatic process [22, 23]. In most of these studies, *in vivo* evidence was obtained in different xenograft models in which immunocompromised mouse host is inoculated with human PDAC cells. In xenograft models in which the PDAC cells are inoculated subcutaneously (animal’s flank) or orthotopically (pancreas), the effects of miRNA activity modulation can be interrogated at several stages of disease progression from growth of the primary tumor to increase migration and invasive behavior to intravasation and extravasation and colonization and growth at distant sites. In xenograft models in which the PDAC cells are inoculated intrasplenically (spleen) or intravenously (tail vein), the effects of miRNA activity modulation can be interrogated at more advance stages of disease progression from extravasation to colonization and growth at distant sites. This *in vivo* evidence suggests that some miRNAs (e.g., miR-200b, miR-323-3p, miR-367, miR-489) appears to exclusively or predominantly influence a specific step of the metastatic process, whereas other miRNAs (e.g., let-7a, miR-10b, miR-21, miR-29b, miR-34a) may influence multiple cellular processes (proliferation, survival, and/or drug resistance) that collectively contribute to tumor progression and metastatic spread.

## miRNA-mediated regulation of epithelial to mesenchymal transition

**Fig. 2:**
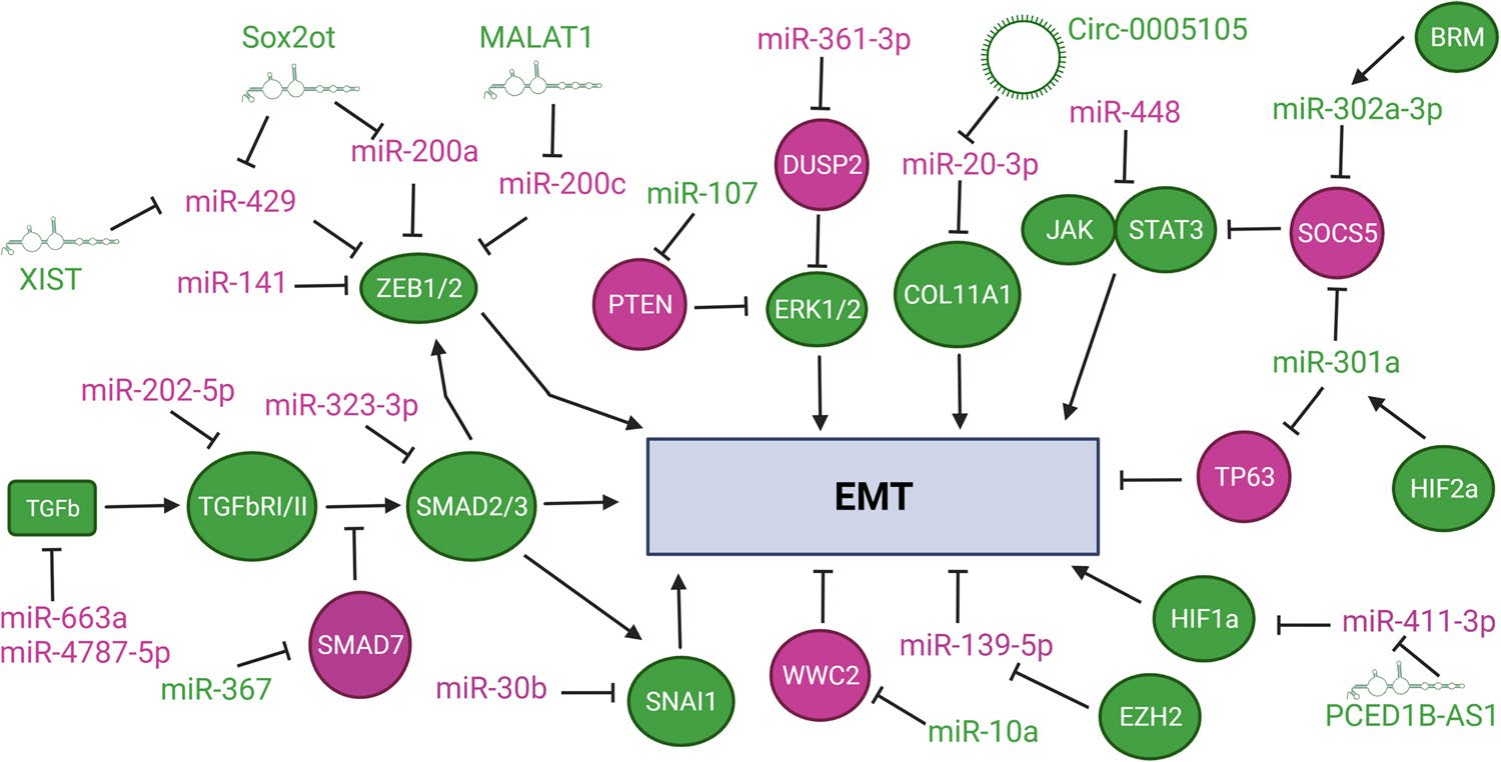
miRNA-mediated regulatory networks influencing EMT program. miRNAs and protein-encoding genes (ovals) depicted in violet maintain or favor epithelial program (anti-EMT, tumor suppressive), whereas miRNAs, lncRNAs, circRNAs, and protein-encoding genes in green favor mesenchymal program (pro-EMT, tumor promoting). In other contexts, these miRNAs may influence other cellular programs and decisions. Abbreviations: BRM, Brahma; COL11A1 collagen 11A1 ; DUSP2, dual specificity phosphatase 2, ERK1/2, extracellular signal-regulated kinases; EZH2, enhancer of zeste 2 polycomb repressive complex 2 subunit; HIF1a/2a, hypoxia inducible factor 1/2 subunit alpha; JAK, Janus kinase; PTEN, phosphatase and tensin homolog; SMAD2/3/7, similar to gene product of *C. elegans* Sma (Small) and *Drosophila* Mad (mothers against decapentaplegic) 2/3/7; SNAI1, snail family transcriptional repressor 1; SOCS5, suppressor of cytokine signaling 5; STAT3, signal transducer and activator of transcription 3; TGFb, tumor growth factor β; TGFbRI/II, TGF-β receptor I/II; TP63, tumor protein 63; ZEB1/2, zinc finger E-box binding homeobox 1/2

Epithelial to mesenchymal transition (EMT) is a process associated with progression and metastatic spread, by which cancer cells acquire increased motility and invasive behavior [26]. TGF-β and HIF signaling are known inducers and regulators of EMT in PDAC [26]. Ultimately, the integration of these and other input signals leads to upregulation of ZEB1/2, TWIST1, and/or SNAI1/2: transcription factors that promote mesenchymal program and downregulation of epithelial cell adhesion protein, E-cadherin. Several miRNAs have been identified in PDAC with a major or predominant role in regulating EMT (Fig. 2; refs [27–46]). miR-202-5p, -323-3p, -663a, and -4787-5p put a break on the EMT program by inhibiting expression of key components of TGF-β signaling pathway [28–30], whereas miR-367 promotes EMT by inhibiting expression of SMAD7, negative regulator of TGF-β signaling [31]. Similarly, miR-301a promotes EMT by relaying hypoxic HIF2α signal to increase JAK1/STAT3 signaling via inhibition of direct target *SOCS5* [32] and to decrease transcription program of direct target *TP63* [33]. miR-302a relays pro-metastatic signal of BRM, a catalytic ATPse subunit of the SWI/SNF chromatin remodeling complex, to increase JAK2/STAT3 signaling also via inhibition of direct target *SOCS5* [39]. In contrast, miR-448 activity suppresses metastatic spread by downregulating expression of direct target gene JAK1 and dampening pro-EMT JAK/STAT3 signaling [40]. miR-10a decreases Hippo signaling and its negative regulation of EMT and cancer cell stemness via inhibition of direct target *WWC2* [46]. Increased activity of WWC2 leads to phosphorylation and activation of LATS1/2, which in turn prevents nuclear translocation and activity YAP/TAZ transcription complex [46]. The miR-200 family members are arranged in two gene clusters (*MIR200b* ~ *MIR200a* ~ *MIR429* at chromosome 1 and *MIR200c* ~ *MIR141* at chromosome 12) in humans. miR-200 family members are considered safeguards of epithelial cell program and identity by inhibiting expression of direct target genes *ZEB1* and *ZEB2* [34–36]. Unlike breast and other cancer types, individual activity of these miRNAs leads to different regulation of EMT and tumor suppressive properties in orthotopic Panc-1 xenograft tumor model [34]. Enforced expression of miR-141 or miR-429 results in a significant inhibition of tumor growth in this orthotopic model, whereas that of other miR-200 family members or their combination with miR-141 and miR-429 does not [34]. miR-141 tumor suppressive and anti-metastatic function is also mediated by direct inhibition of MAP4K4 [37] and WIPF1 [38], respectively. There is a differential association between expression changes of individual miR-200 family members and that of ZEB1, ZEB2, and E-cadherin in PDAC clinical samples and patient-derived xenograft tumors [34]. In a cohort of 31 PDAC primary tumors, there is no consistent trend of downregulation for all miR-200 family members, but there is a positive correlation between expression of all miR-200 family and that of E-cadherin [34]. Negative association is only statistically significant for expression of miR-200a and miR-141 in relation to that of *ZEB1* and *ZEB2* [34]. These results add a level of complexity to the regulation of EMT and perhaps other metastatic processes by each of these related miRNAs and suggest the importance of context, timing, and differential inhibition of key target genes (other than *ZEB1*/*ZEB2*).

## miRNA-mediated regulation of extracellular matrix remodeling, invasion, and metastatic spread

**Fig. 3:**
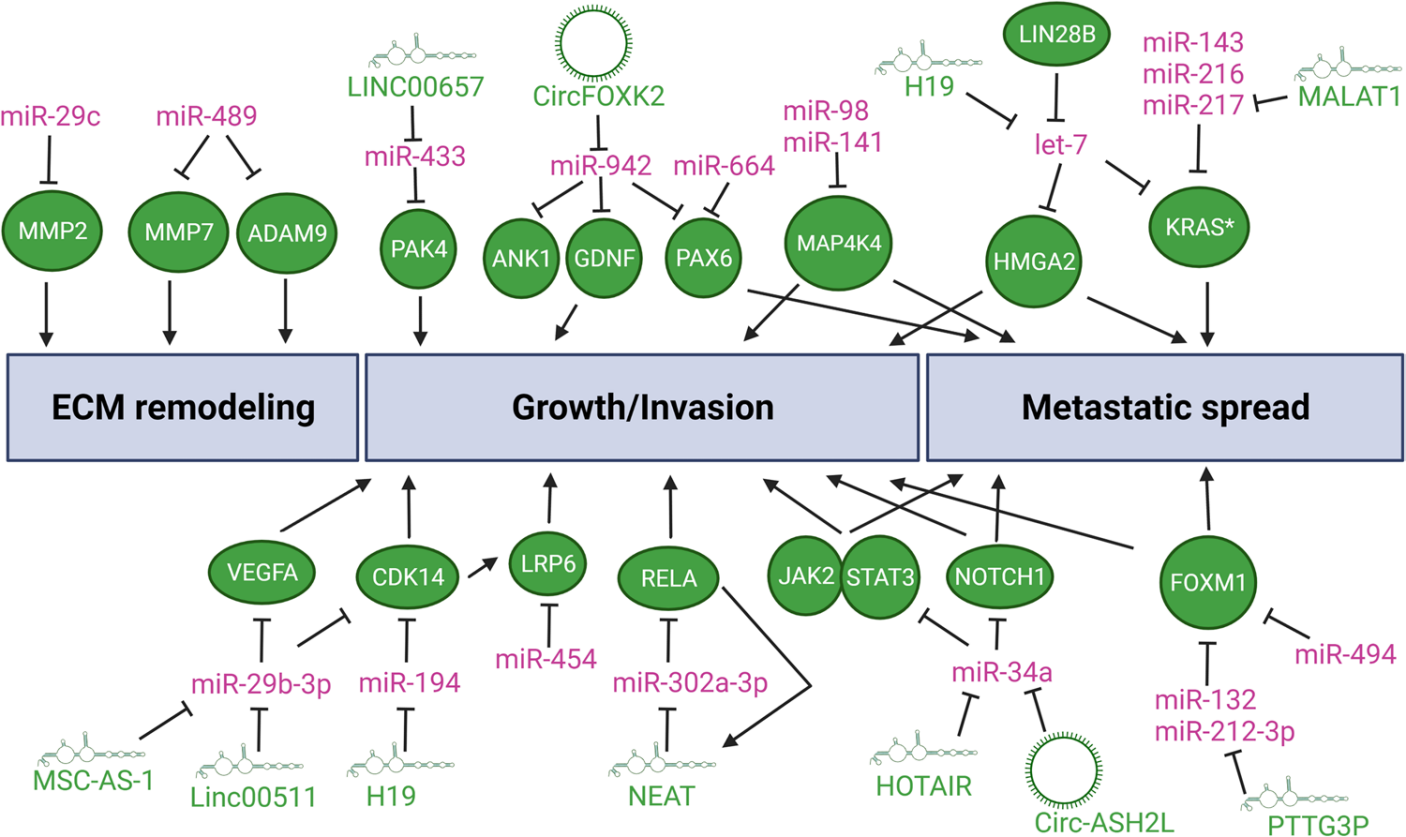
miRNA-mediated regulatory networks influencing metastatic programs. miRNAs depicted in violet act as a tumor suppressive and/or anti-metastatic factor whereas lncRNAs, circRNAs, and protein-encoding genes (ovals) in green as tumor promoting and/or pro-metastatic factors. In other contexts, these miRNAs may influence other cellular programs and decisions. Abbreviations: ADAM9, A disintegrin and metalloproteinase domain 9; ANK1, ankyrin 1; CDK14, cyclin-dependent kinase 14; FOXM1, forkhead box M1; GDNF, glial cell derived neurotrophic factor; HMGA2, high mobility group AT-Hook 2; JAK2, Janus kinase 2; KRAS*, mutant KRAS Proto-Oncogene, GTPase; LIN28B, Lin-28 homolog B; LRP6, LDL receptor related protein 6; MAP4K4, mitogen-activated protein kinase kinase kinase kinase 4; MMP2/9, matrix metallopeptidase 2/9; NOTCH1, notch receptor 1; PAK4, P21 (RAC1) activated kinase 4; PAX6, paired box 6; RELA, RELA Proto-Oncogene, NF-κB Subunit; STAT3, signal transducer and activator of transcription 3; VEGFA, vascular endothelial growth factor A

The extracellular matrix (ECM) is composed proteoglycans, proteins, and matricellular associated proteins. ECM remodeling promotes growth, survival, and invasion of PDAC cells at the primary tumor and facilitates colonization at premetastatic niches in the liver and other distant organs [47]. Some miRNAs have a direct role in limiting the tumorigenic remodeling of the ECM (Fig. 3, refs [48, 49]) by modulating the expression of matrix metalloproteinases (MMPs) and disintegrin and metalloproteinases (ADAMs). miR-29c activity inhibits the expression of direct target gene *MMP2* [48]. Sequential selection of Hs766t cells with preferential metastasis to the liver shows the requirement of miR-29c to limit metastatic spread [48]. It is not clear if miR-29c is more crucial at escaping the primary tumor site and/or reducing colonization and growth at distant metastatic site. miR-489 activity inhibits the expression of direct target genes *MMP7* and *ADAM9* and is required for limiting colonization and growth at distant metastatic sites [49]. Other miRNAs influence cellular processes of growth, invasion, and/or metastatic spread that indirectly affect cancer cell-mediated remodeling of the ECM, interaction with ECM elements, and/or mechanosensing of stroma stiffness (Fig. 3, refs [27, 50–65]). miR-664 and miR-942 modulate cancer cell growth and invasion by direct inhibition of common target gene transcription factor *PAX6* [53, 54]. Similarly, miR-132, miR-212-3p, and miR-494 modulate invasion and metastatic spread by direct inhibition of common target gene transcription factor *FOXM1* [55, 56]. SMAD4 positively regulates expression of miR-494 via canonical TGF-β signaling pathway [56]. Genetic loss of *SMAD4* downregulates miR-494 expression and leads to upregulation of FOXM1 along with potentiation of WNT signaling by FOXM1-mediated nuclear translocation of *β*-catenin [56]*.* This illustrates the complexity and duality of TFG-β signaling in suppressing tumor growth in early stages of carcinogenesis while promoting tumor growth and metastasis via EMT and immunosuppression (most of the other cases in this review) in later stages. Gene-dosage increases of mutant *KRAS* alleles drive early stages of carcinogenesis and later metastatic spread [14]. Several miRNAs, including let-7a, miR-143, miR-216, and miR-217, directly bind to the 3′UTR of both wild-type and mutant *KRAS* mRNAs [27, 57–65]. Overcoming this miRNA-mediated regulation of KRAS expression may also contribute to the mutant KRAS dosage-dependent switch to tumor progression and metastatic spread. let-7a, a founding member of the miRNA gene family, is considered a potent tumor suppressor gene. Other targets of let-7 include potent oncogenic factors such as c-MYC and HMGA2 [27, 64, 65]. By coordinately regulating the expression of KRAS, HMGA2, and c-MYC, let-7 activity restrains cell proliferation, cell cycle progression, invasion, EMT, and metastasis. LIN28B is an oncofetal RNA-binding protein that interferes with the maturation process of let-7 precursor RNA and in so doing increases oncogenic expression of these let-7 key target genes in PDAC [64, 65]. LIN28B is highly expressed in circulating tumor cells of PDAC patients and is a driver of metastatic dissemination [64]. CRISPR-mediated knockout of *LIN28B* gene, chemical inhibition of LIN28B binding to let-7, or knockdown of HMGA2 significantly diminish the metastatic potential of circulating tumor cells [64]. This indicates the crucial role of let-7 in suppressing LIN28B-depedent metastatic program in PDAC.

## Interactions between miRNAs and other non-coding RNAs dictate metastatic programs

An emerging layer of regulation in many miRNA/key target pathways in PDAC is that of non-coding RNA dampening miRNA activity. While miRNAs are a well-defined class of short ~ 22 nts RNAs, longer non-coding RNAs are more diverse and complex classes of RNAs (Fig. 1). Long non-coding RNAs (lncRNAs) are a heterogenous class that broadly includes a variety of non-coding RNAs longer than 200 nts [66]. lncRNAs can act as local regulators of transcription by interacting with epigenetic machinery, squelching, or blocking access of transcription factors; provide scaffolding structure for RNA-RNA and RNA–protein interactions; and bind and sequester miRNA molecules [66–68]. Circular non-coding RNAs (circRNAs) are covalently closed loops resulting from backsplicing of mRNAs [67, 69]. The main role of circRNAs is to bind and act as sponges of miRNAs [67, 69]. We describe below a few examples of these miRNA-ncRNA interactions in regulatory pathways pertinent to tumor progression and metastatic processes already highlighted in previous sections (Figs. 2 and 3, refs [27, 35, 36, 54, 70–78]). lncRNA MALAT1 binds to miR-200c and sequesters it away from binding sites on the 3′UTR of *ZEB1*/*ZEB2* mRNAs and other target genes, thereby skewing cellular program towards EMT [36]. Similarly, lncRNA Sox2ot and XIST promote EMT by sequestering other miR-200 family members (miR-200a and miR-429) [35, 72]. This differential interaction and modulation of specific miR-200 family members by these lncRNA may explain, in part, discordant results of *in vivo* functional analysis of each family member [34]. lncRNA PCED1B-AS1 potentiates HIF-mediated EMT by sequestering miR-411-3p which directly inhibits expression of HIF1α [73]. lncRNA MSC-AS-1 sequesters miR-29b, a family member of EMT-regulating miR-29c, which leads to upregulation of cyclin-dependent kinase CDK14 [74]. lncRNA H19 sequesters miR-194, which also leads to upregulation of CDK14 [75]. CDK14 activates WNT signaling by phosphorylating the LRP5/6 receptors during G2/M phase of the cell cycle. Conversely, miR-454 inhibits expression of LRP6 and dampens WNT signaling [50]. This suggests a converging network of lncRNA-miRNA interactions that modulate cell cycle-dependent WNT signaling. H19 also sequesters let-7a and interferes with its ability to inhibit HMGA2-mediated EMT program [27].

miR-34a is a potent tumor suppressive miRNA that can concomitantly inhibit expression of several proto-oncogenic genes (e.g., BCL2 and c-MET) and signaling pathways (e.g., NOTCH, WNT) [79]. lncRNA HOTAIR sequesters miR-34a and leads to activation of JAK2/STAT3 signaling pathway [77]. By interfering with miR-34a activity, HOTAIR promotes cancer cell stemness, EMT, and metastatic spread of PDAC cells [77]. circRNA Circ-ASH2L sequesters miR-34a what leads to upregulation of miR-34a–direct target gene *NOTCH1* [76]. Circ-ASH2L-mediated activation of NOTCH signaling pathway promotes angiogenesis, tumor growth, and tumor invasion [76]. Another circRNA, CircFOXK2, also promotes tumor growth and metastasis by sequestering miR-942 and upregulating expression of its direct target genes *ANK1*, *GDNF*, and *PAX6* [54]. CircFOXK2 may also influence these processes in a miR-942-independent manner by interacting with proteins involved in mRNA splicing, YBX1 and hnRNPK, which leads to upregulation of NUF2 and PDXK oncogenic expression [54]. There are additional RNA-RNA interactions of metastasis-modulating miRNAs and other ncRNA classes (e.g., lncRNA TUG1-miR-29c, lncRNA DUXAP8-miR-488) in PDAC [80–82]. Although these interactions have been reported in the context of other regulatory pathways and/or cellular processes, they may also contribute to metastatic programs.

## microRNA-mediated cellular crosstalk influences metastatic processes

**Fig. 4:**
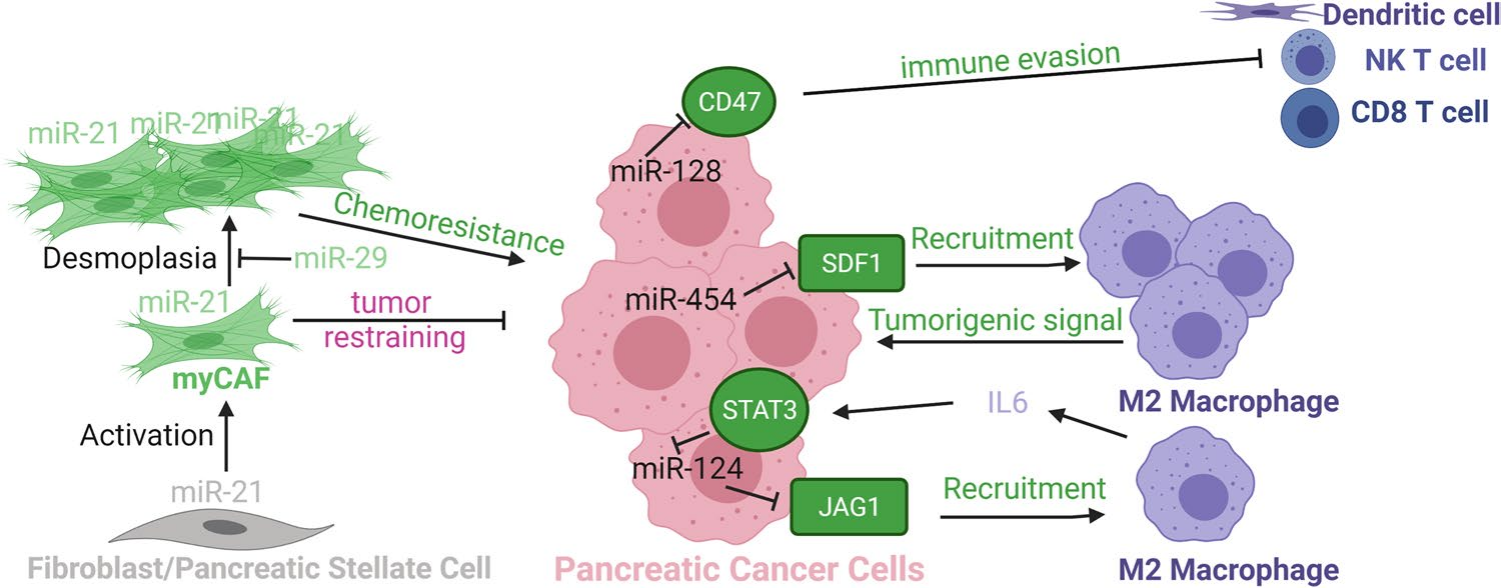
miRNA-mediated cellular crosstalk influencing metastatic programs. The color of the miRNA indicates the identity of the expressing cell type. Proteins (ovals and round-edge rectangles for ligands) and processes in green depict a pro-metastatic role and process in violet an anti-metastatic role. In other contexts, these miRNAs may influence other cellular programs and decisions. Abbreviations: CD8, T-cell surface glycoprotein CD8 alpha chain; CD47, leukocyte surface antigen CD47; IL6, interleukin 6; JAG1, jagged canonical Notch ligand 1; myCAF, myofibroblastic cancer-associated fibroblast; NK T cell, Natural Killer T cell; SDF1, stroma derived factor 1; STAT3, signal transducer and activator of transcription 3

It is well established that cancer cell-stroma interactions in PDAC have an influence on chemoresistance, disease progression, and metastatic spread. Several miRNAs have been implicated in this crosstalk between cancer cells and other cell types in the TME (Fig. 4; refs [83–88]). In some instances, miRNA-mediated regulation in cancer cells leads to a receptor/ligand-mediated response in another cell type of the TME that may then lead to a paracrine signal back to the cancer cells or other cell types in the TME. In other instances, miRNA-mediated regulation in cancer-associated fibroblasts (CAFs) or other cell types in the TME leads to physical interaction and/or paracrine signal that affects cancer cell growth and treatment resistance. miR-124 is engaged in a regulatory feedback loop with NOTCH signaling pathway in cancer cells and crosstalk with tumor-associated macrophages (TAMs). miR-124 inhibits metastatic program by directly targeting JAG1 in cancer cells dampening NOTCH signaling [83]. Increased NOTCH signaling recruits and polarizes TAMs to a tumorigenic M2 phenotype [83]. IL-6 secreted by these M2 TAMs upregulates STAT3 signaling in cancer cells, which downregulates miR-124 expression and promotes EMT program [83]. Similarly, cancer cell–expressed miR-454 inhibits the expression of stromal cell derived factor 1 (SDF1, also known as chemokine CXCL12) [84]. Increased SDF1 levels recruit TAMs via activation of their chemokine receptor CXCR4, creating a pro-tumorigenic TME [84]. In contrast, cancer cell–expressed miR-128 inhibits metastatic program by directly targeting ZEB1, a positive regulator of EMT and CD47-mediated tumor immune evasion [85]. Enforced expression of miR-128 in orthotopic syngeneic Panc02 cell model dampens CD47 expression and thereby increases anti-tumor immunity mediated by dendritic cells, CD8 + , and Natural Killer T cells [85].

miR-21 is generally considered an oncogenic miRNA and important mediator of TGF-β-induced EMT via inhibition of *PTEN*, *PDCD4*, and/or *RECK* expression [89]. However, miR-21 also responds to these inputs and regulates these and other direct targets in other cellular elements of the TME, including CAFs and TAMs [18, 90]. New 3D co-culture techniques, single-cell RNAseq, and mechanistic studies in GEMMs have uncovered the existence of distinct CAF subtypes in PDAC [91, 92]. Low or high expression of α-smooth muscle actin (SMA) is a consistent differential feature of CAF subtypes; “myofibroblastic” CAFs (myCAFs) express high levels of SMA, are juxtaposed or in close proximity to cancer cells, and exhibit tumor-restraining function [93–99]. Co-detection of miR-21 and SMA expression demonstrates a predominant upregulation of miR-21 expression in myCAFs in the majority of PDAC tumors from patients and GEMMs [86, 87, 100–107]. myCAF expression of miR-21 provides more robust prognostic information than epithelial expression for predicting treatment response and overall survival of PDAC patients [87, 104, 105]. *In vitro* and *in vivo* co-culture assays of cancer cells and fibroblasts/CAFs suggest a CAF-driven role of miR-21 [87, 104, 108]. Inhibition of miR-21 activity in CAFs before co-inoculation with cancer cells enhances efficacy of gemcitabine in a subcutaneous syngeneic Pan02 cell model [87]. Strikingly, global loss of miR-21 activity accelerates tumor development and results in a much shorter overall survival in a mutant K-Ras p53-deleted PDAC GEMM (KPC model) [86]. The absence of myCAFs in precursor and invasive lesions is the most outstanding consequence of miR-21 loss. Cancer cell–specific activity of miR-21 is not required for *in vitro* or *in vivo* growth nor affects *in vitro* chemoresistance to gemcitabine in this KPC model [86]. This study uncovers a pro-fibrotic yet tumor-restraining activity of miR-21 suggestive of a cell-intrinsic myCAF role. Timing, duration, and extent of miR-21 inhibition, mutational landscape, and/or tumor subtype may help explain the discrepancies between studies (Fig. 4). Interestingly, expression of miR-29a and miR-29b is downregulated in pancreatic stellate cells/resting fibroblasts during TGF-β-induced pancreatic stellate cell/CAF activation [88]. miR-29 activity appears to limit ECM protein accumulation and desmoplastic reaction via direct inhibition of collagen (*COL1A1*, *COL3A1*) and laminin (*LAMC1*) mRNAs [88]. Understanding how these miR-21–regulated and miR-29–regulated processes interfere with each other to affect myCAF function and composition of other CAF subtypes under chemotherapy treatment is a key question to answer in order to achieve more effective and durable clinical responses.

## microRNA-mediated pro-metastatic cell-to-cell communication via extracellular vesicles

**Fig. 5:**
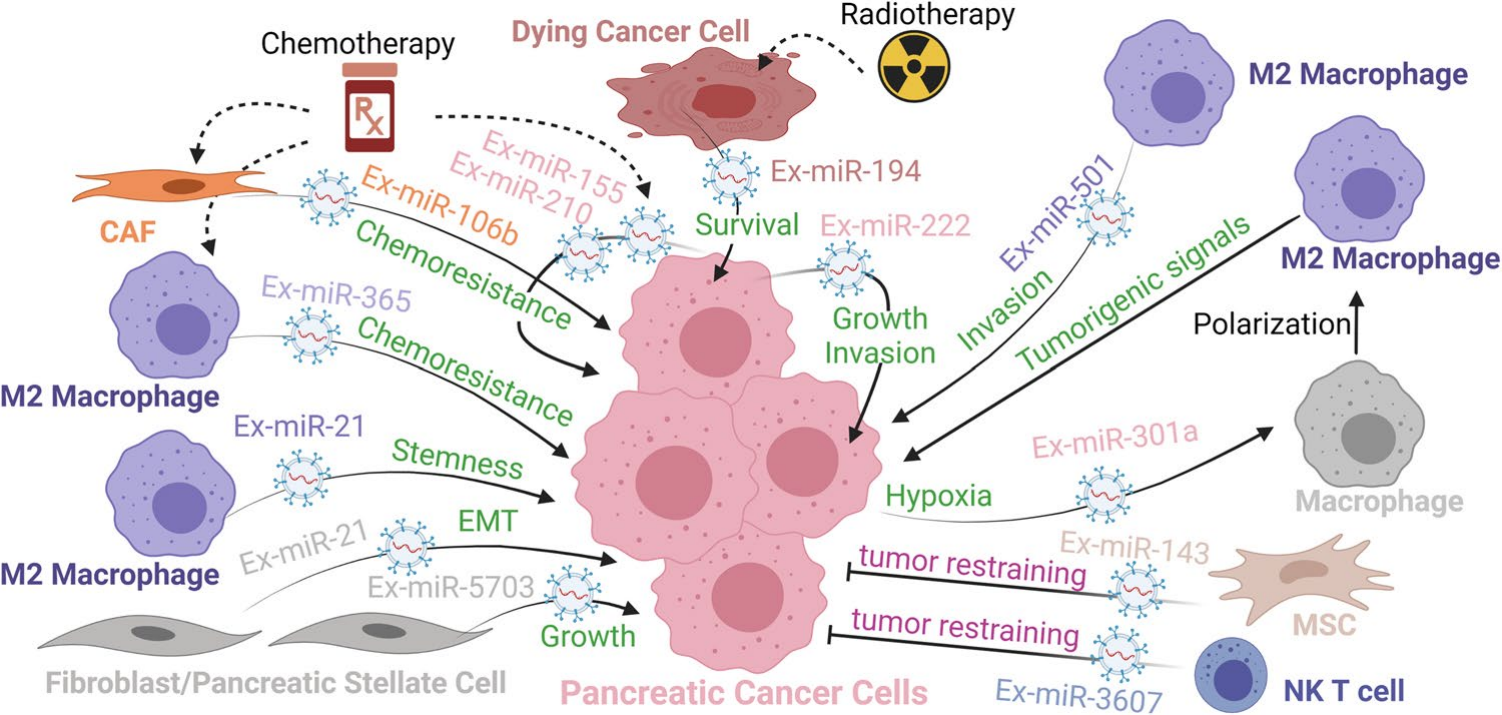
microRNA-mediated cell-to-cell communication via extracellular vesicles. The color of the Ex-miRNA indicates the identity of the cell type secreting the EV-bound miRNA. Processes in violet depict an anti-metastatic role whereas those in green a pro-metastatic role. In other contexts, these Ex-miRNAs may influence other cellular programs and decisions. Abbreviations: CAF, cancer-associated fibroblast; MSC, mesenchymal stem cell; NK T cell, Natural Killer T cell

miRNAs themselves can be the regulatory signal from one cell type to another via transfer of extracellular vesicles (EVs). There are a few classes of membrane-bound EVs depending on their origin and size that mediate in cell-to-cell communications: microparticles, shed microvesicles, or ectosomes are directly formed by budding of the cell plasma membrane and range in size 10 nm to 3 μm; exosomes are secreted via multivesicular-body endocytic process and are about 100 nm in size (40–150 nm range). Because there are technical and methodological challenges in the purification of exosomes from a mixed population of other classes of secreted vesicles [23, 109–111], we will use the more general term of EV in accordance with the International Society of Extracellular Vesicles [112] even though the original research study may have used the term exosome. We will refer to the miRNA loaded and transferred in EVs from one cell to another as Ex-miRNA. In some instance, the Ex-miRNA can act as a hormone-like signal and interact with RNA sensing receptors such as Toll-like receptor 8 [113], but generally the transferred Ex-miRNA is loaded to miRISC and controls gene expression at the posttranscriptional level in the recipient cells [23, 109, 111]. Ex-miRNAs can be secreted from cancer cells in response to treatment, hypoxia, or other inputs in the TME to affect cellular processes at a distance; recipient cells may be other cancer cells or other cell types in the TME (Fig. 5, refs [114–117]). Similarly, ex-miRNAs can be secreted from CAFs, TAMs, and other cell types in the TME to affect a variety of cellular processes in the recipient cancer cells (Fig. 5, [61, 118–125]). Ex-miR-222 secreted from more aggressive and metastasis-prone cancer cells can stimulate proliferative and invasion program in neighboring cancer cells by downregulating the expression and activity of cyclin-dependent kinase inhibitor p27 [114]. miR-222 directly binds and inhibits *p27* mRNA and indirectly increases AKT-mediated phosphorylation and cytoplasmic translocation of p27 via inhibition of serine/threonine-protein phosphate PPP2R2A expression [114]. Radiotherapy induces the secretion of Ex-miR-194-5p from dying cancer cells [115]. Ex-miR-194-5p potentiates the survival of neighbor cancer cells, especially those with stem cell–like and tumor repopulating properties, by inducing a temporary G1/S arrest and upregulation of DNA damage response via direct inhibition of E2F3 and HMGA2 [115]. Latent release of prostaglandin E2 from dying cells stimulates proliferation of Ex-miR-194-5p–protected cancer cells. Combining radiotherapy with low-dose aspirin enhances treatment efficacy by reducing both the amount of Ex-miR-194-5 pm and prostaglandin E2 released from dying cells [115]. Similarly, chemotherapy treatment with gemcitabine induces the secretion of Ex-miR-155 and Ex-miR-210 from chemosensitive cancer cells, which leads to increase chemoresistance in recipient cancer cells [116, 117]. Higher levels of miR-210 in recipient cells activate mTOR pathway, though the direct key target genes of miR-210 leading to this activation have not been identified. Gemcitabine also induces the secretion of Ex-miR-106b from CAFs. In recipient cancer cells, miR-106b enhances chemoresistance via direct inhibition of *TP3INP1* expression [119], a stress-induced p53-target gene with anti-proliferative and pro-apoptotic activity. M2-polarized TAMs secrete Ex-miR365 that contributes to gemcitabine chemoresistance in cancer cells by augmenting the gemcitabine-competing triphospho-nucleotide pool and upregulating gemcitabine-inactivating cytidine deaminase [120]. Direct target genes that miR-365 regulates in the process have not been identified.

Hypoxic TME induces HIF-dependent release of Ex-miR-301a from cancer cells to TAMs [121]. In TAMs, miR-301a upregulates PI3Kγ signaling pathway via direct inhibition of PTEN expression, which favors polarization to tumorigenic M2 phenotype [121]. These M2 TAMs secrete TGF-β, IL-10, and arginase that promote cancer cell metastasis and potentiate miR-301a-mediated EMT program as described above (Fig. 2). While it is not clear if in direct response to Ex-miR-301a transfer, M2 TAMs can secrete Ex-miR-21 and Ex-miR-501 that are taken up by cancer cells. In cancer cells, miR-501-3p downregulates expression of TGFβR3, whose shedded ectodomain curtails TGF-β signaling, to favor migration and invasive behavior [122]. In cancer cells, miR-21 downregulates the expression of transcription factor KLF3 to promote NANOG/OCT4-dependent cancer cell stemness program [123]. Pancreatic stellate cells secrete Ex-miR-21 which in recipient cancer cells activates PI3K/AKT pathway and promotes EMT program and MMP2/9-mediated ECM remodeling [124]. Pancreatic stellate cells also secrete Ex-miR-5703, which in recipient cancer cells activates PI3/AKT pathway and promotes cell proliferation via downregulation of CMTM4 [125]. While beyond the scope of this review, there is growing evidence of cell-to-cell communication via secreted lncRNAs and circRNAs that inhibit miRNA activity in recipient cells in PDAC tumors [35, 126]. This adds another exciting and complex regulatory layer to the field of RNA-RNA interactions and endogenous competing RNA hypothesis.

## Pharmacological interventions to modulate miRNA activity

Several therapeutics strategies to modulate the activity of a specific miRNA with synthetic RNA analogs and different delivery technologies have been tested in preclinical models and some are being investigated in clinical trials [18, 127, 128]. An attractive feature of miRNAs as drug target is that activity modulation of a single miRNA could have a broad influence in several direct targets and processes downstream of these regulatory interactions. A potential advantage of a miRNA drug is that it could be more directed to a particular cell type and/or have a more restricted effect on selected cellular processes than small molecule inhibitors targeting upstream signaling pathways such TGF-β, JAK/STAT, or PI3K/AKT. There are several technical considerations for the chemical modifications and delivery approaches when replenishing the activity of tumor suppressive and/or anti-metastatic miRNA vs. inhibiting the activity of a tumor promoting and/or pro-metastatic miRNA [18, 127, 128]. miRNA mimics to replenish miRNA activity are typically double-stranded modified RNA analogs that need to be taken up by cells, released in an intact form in the cytoplasm for DICER processing, miRISC loading and binding to target mRNAs, to be functionally active. In contrast, anti-miRNA inhibitors to inhibit miRNA activity are typically single-stranded modified RNA analogs that need to be taken up by cells and then released in the cytoplasm to bind to perfectly complementary miRNA molecules. Thus, the design and delivery of a miRNA mimic is more challenging than that of an anti-miRNA inhibitor [18, 128]. miRNA mimics require encapsulation in liposomal nanoparticles or complexing with polymers or other coated nanoparticles [18, 128]. A double-stranded miR-217 mimic encapsulated in a PEGylated lipid nanoparticle, decorated with iRDG tumor penetrating–peptide, is effective at downregulating KRAS expression *in vitro* cell assays, but it may not be as effective as short interfering RNAs (siRNAs) against *KRAS* in an *in vivo* subcutaneous PDAC tumor model [129]. miR-34a is an excellent candidate for replenishing therapy in PDAC [130]. A miR-34a-expressing DNA vector encapsulated in liposomal nanoparticles (nanovector) is effective at reducing tumor growth in subcutaneous and orthotopic xenograft models, and more so than similar nanovector strategy with *KRAS*-targeting miR-143 and miR-145 [130]. Forced expression of miR-34a significantly reduces tumor expression of direct target genes (*CD44*, *ALDH*) involved in cancer cell stemness [130]. miR-34 mimetic drug, MRX34, was the first miRNA replacement therapy to enter clinical trials for treatment of primary liver tumors or cases with liver metastasis [18]. Unfortunately, on-target adverse immunological effects caused termination of these clinical trials [131]. Re-formulation of the liposomal nanoparticle or use of other platforms that may preferentially deliver the miR-34 mimic to cancer cells could bypass this immune toxicity. A poly (D,L-lactide-co-glycolide) (PLGA)-based nanoparticle formulation has a high encapsulation capacity for and effectively can delivery miRNA mimics to PDAC cell lines *in vitro* [132]. While this nanoparticle formulation was only tested for a miR-150 mimic and its interaction with direct target *MUC4* [132], it may be a viable and low toxicity option for systemic delivery of a miR-34 mimic. Natural or synthetic exosomes may also be used as an encapsulation and delivery vehicle to modulate miRNA activity at primary or metastatic tumor sites in PDAC [133–137]. In comparison to other nanoparticle formulation (e.g., liposomes, micelles, inorganic nanoparticles), exosomes can have a greater drug delivery potential due to their lower clearance rate, deeper tissue penetration, and enhanced biocompatibility [138, 139]. Ultrasound-assisted loading of miR-34a mimic molecules in 293 T-derived exosomes provides a promising delivery platform to inhibit cancer cell growth in an *in vivo* xenograft PDAC model [140]. In this study, miR-34-loaded exosomes effectively downregulate expression of anti-apoptotic BCL2 in the xenograft tumors [140]. This or a similar strategy could be considered for investigating miR-34 replacement therapy in PDAC patients.

Anti-miRNA inhibitors can tolerate more chemical modifications since they do not require processing by the cellular machinery. Anti-miRNA inhibitors can be delivered systemically without the need of encapsulation, conjugation, or complexing, though these can offer intrinsic imaging and/or more targeted delivery capabilities. Administration of an unconjugated heavily modified single-stranded anti-miR-21 antisense oligonucleotide in the K-Ras-driven p53-mutated KPC model at an early age is remarkably effective at preventing tumor progression from pancreatic intraductal precursor lesion to malignant invasive carcinoma in this aggressive GEMM [141]. As we described above, miR-21 is a multifaceted miRNA with functional activity in different cell types. An important consideration for clinical evaluation of anti-miR-21-based cancer interception strategy will be to understand if a specific cell type or multiple ones are driving malignancy. A similar anti-miR-21 inhibitor encapsulated in PEGylated lipid nanoparticle, decorated with iRDG tumor penetrating–peptide and/or transportan cell-penetrating–peptide, reduces tumor growth in patient-derived and organoid-derived xenograft models [129, 142]. This therapy appears to be effective only in cancer cell–derived models with high levels of miR-21 at baseline, suggesting an intertumoral heterogeneity in terms of which miR-21-expressing cell types contribute to malignancy. Expression and function of miR-10 family members, miR-10a and miR-10b, have been associated with pro-metastatic programs in PDAC [46, 143–148]. In glioblastoma and breast cancer models, several encapsulation and nanoparticle strategies, including dextran-coated iron oxide nanoparticles, have been successful at delivering anti-miR-10b modified oligonucleotides to primary and metastatic tumor sites and causing miR-10b-dependent growth inhibition [18]. A similar dextran-coated iron oxide nanoparticle platform was recently used to deliver siRNAs against immune checkpoint PD-L1 in an orthotopic Pan02 syngeneic model of PDAC [149]. Thus, this nanoparticle platform with intrinsic magnetic resonance imaging capability could be applied to investigate the therapeutic efficacy of anti-miR-10b *in vivo* models of PDAC.

## Conclusions

The level of evidence and biological effects of these discussed miRNAs and RNA-RNA networks varies depending on experimental design, model used, and clinical validation. By design, most studies focus on a particular miRNA-mediated process, but all these miRNA-regulated processes may be occurring at once in a PDAC tumor. What the cumulative effects of these miRNAs and RNA-RNA networks are and which of these miRNA-regulated processes may have a more impactful contribution to tumor progression and metastatic spread and in what cell types are still important remaining questions. Answering these questions should guide prioritizing of clinical development of miRNA targeting strategies that would be most beneficial for PDAC patients.
